# Anaesthetic Management of a Patient With a Giant Ovarian Tumour Containing 28 Litres of Ascitic Fluid

**DOI:** 10.7759/cureus.101824

**Published:** 2026-01-19

**Authors:** Rahul Kalshan, Trisha Nidhi

**Affiliations:** 1 Anaesthesia, Safdarjung Hospital, New Delhi, IND

**Keywords:** dyspnoea, epidural analgesia, general anaesthesia, giant ovarian tumour, massive ascites

## Abstract

We report the anaesthetic management of a 47-year-old woman scheduled for tumour resection of a giant ovarian tumour containing 28 litres of ascitic fluid. Her preoperative abdominal circumference was 186 cm due to massive ascites, and metabolic equivalents (METs) were less than 3.5. She was planned for cytoreductive surgery under general anaesthesia combined with epidural analgesia. Anaesthetic challenges included ventilatory management to maintain airway pressures, haemodynamic stability during massive fluid shifts, extensive monitoring, pre-induction epidural catheter placement, prevention of hypothermia, correction of metabolic or electrolyte derangements, and adequate postoperative pain relief.

## Introduction

Ovarian cancer (OC) is the leading cause of mortality among gynaecological cancers in developed countries and the fifth leading cause of cancer-related mortality globally in women [1]. Ascites is the most common presenting symptom in patients with ovarian carcinoma [2]. More than two-thirds of patients present in advanced stages (III and IV), when ascites leads to abdominal distension, dyspnoea, weight gain, lower extremity oedema, nausea, and vomiting. The reported survival rate in advanced disease ranges from 5% to 20% [3].

Patients with giant ovarian tumours are at increased risk of perioperative complications and require meticulous anaesthetic management. Prior to tumour removal, supine hypotensive syndrome and ventilatory compromise may occur due to mass effect. Following tumour decompression, a sudden reduction in intra-thoracic pressure may precipitate haemodynamic instability and re-expansion pulmonary oedema [4].

## Case presentation

A 47-year-old woman (height 154 cm and weight 80 kg) with a giant ovarian tumour was scheduled for tumour resection. She was a known case of hypertension and hypothyroidism and was on regular medications. Her preoperative abdominal circumference was 186 cm. Her body mass index (BMI) was 33.6 kg/m^2^ (obesity class I) [5]. She experienced dyspnoea during routine physical activity and was comfortable only at rest. Her functional capacity was poor, with metabolic equivalents (METs) less than 3.5 [6].

**Figure 1:**
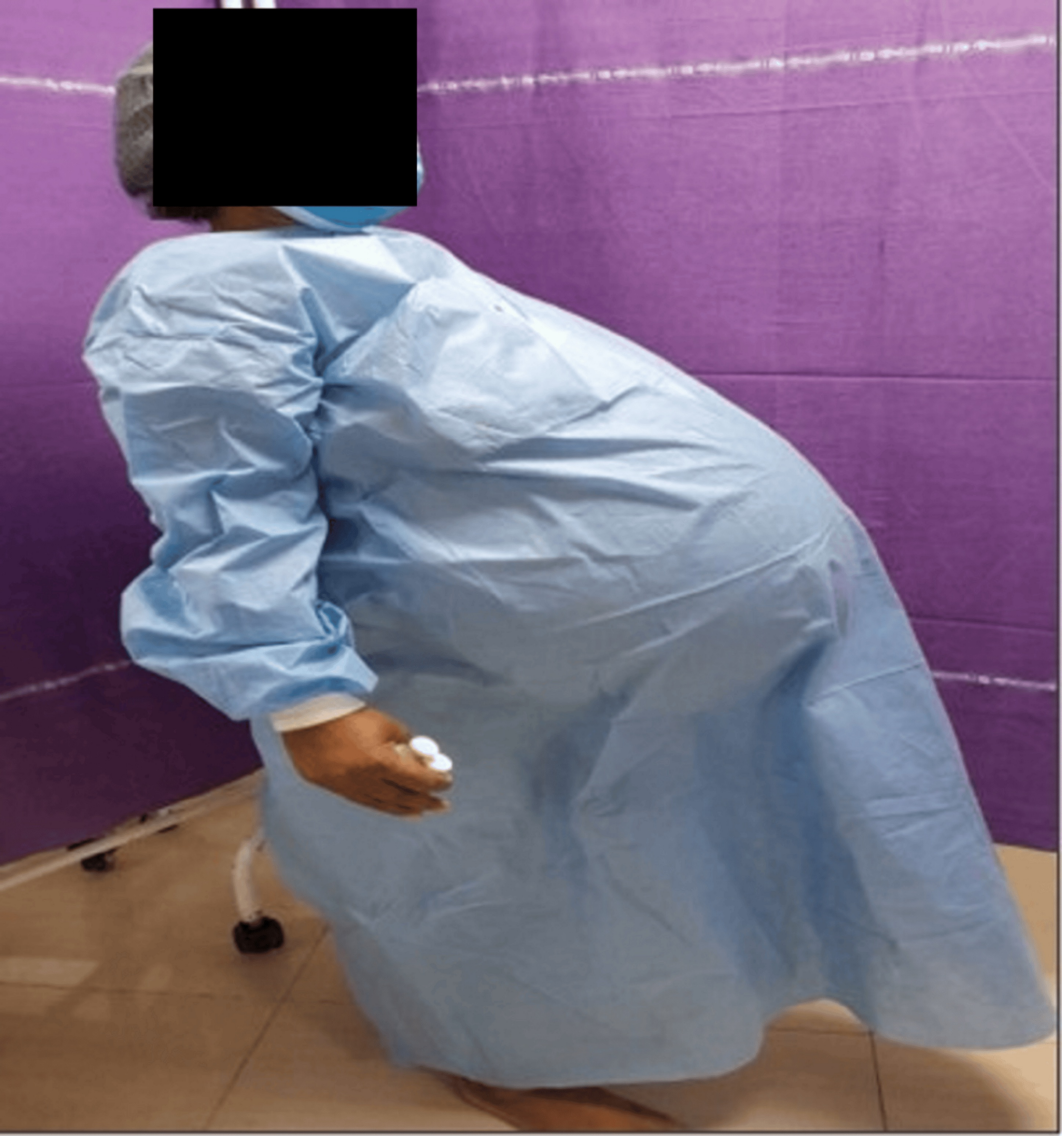
Preoperative photograph showing severe abdominal distension due to massive ascites secondary to a giant ovarian tumour.

She had undergone repeated paracentesis for refractory ascites every two to three months for the past one and a half years. Preoperative clinical examination revealed marked abdominal distension secondary to massive ascites (Figure 1).

18F-fluorodeoxyglucose positron emission tomography-computed tomography (18F-FDG PET-CT) revealed bilateral metabolically active adnexal solid cystic masses with intra-abdominal lymphadenopathy, omental and peritoneal deposits, and gross ascites.

Airway examination revealed Mallampati grade II [7]. Auscultation showed reduced breath sounds at the right lung base. Pulse oximetry demonstrated an oxygen saturation of 97% on room air, and electrocardiography was normal. Chest radiography showed a small right-sided hydrothorax with blunting of the costophrenic angle. Transthoracic echocardiography demonstrated good cardiac function with an ejection fraction of 68%. Pulmonary function testing could not be performed. Arterial blood gas analysis was within normal limits. Laboratory investigations revealed a serum albumin of 3.9 g/dL, potassium of 4.4 mmol/L, normal renal function, and normal coagulation parameters. Preoperative haemoglobin was 13.3 g/dL (Table 1).

**Table 1:** Preoperative laboratory investigations

Parameter	Patient Value	Normal Range
Haemoglobin (g/dL)	13.3	12-15
Serum albumin (g/dL)	3.9	3.5-5.0
Potassium (mmol/L)	4.4	3.5-5.1
Serum creatinine (mg/dL)	0.8	0.6-1.2
Prothrombin time (sec)	12.1	11-13.5
International normalised ratio (INR)	1.0	0.8-1.2
Arterial blood gas	Within normal limits	-

On the day of surgery, the patient was shifted to the operating theatre after obtaining high-risk informed consent and ensuring the availability of blood products.

Standard monitoring (SpO_2_, ECG, non-invasive blood pressure, temperature, end-tidal CO_2_, and neuromuscular monitoring) was applied. Venous access was secured with two 18-gauge intravenous cannulas. Initial vital signs were blood pressure 110/70 mmHg, heart rate 90 beats/min, and SpO_2_ 100% on room air. An epidural catheter was inserted at the L2-L3 interspace under local anaesthesia in the sitting position.

General anaesthesia was induced in the ramp position. After 10 minutes of preoxygenation and infusion of 1000 mL crystalloid, a modified rapid sequence induction was performed using propofol 100 mg, rocuronium 40 mg, and fentanyl 100 µg. Tracheal intubation was achieved on the first attempt using a 7.5-mm cuffed endotracheal tube. Mechanical ventilation was initiated with a tidal volume of 400 mL, a respiratory rate of 15/min, and a positive end-expiratory pressure (PEEP) of 5 cm H_2_O. Anaesthesia was maintained with oxygen, nitrous oxide, and sevoflurane. Neuromuscular blockade was guided by monitoring, and fentanyl was administered as required. Epidural analgesia was activated using a test dose of 3 mL of 2% lignocaine with adrenaline, followed by graded doses of 0.25% bupivacaine.

The left radial artery was cannulated for continuous invasive blood pressure monitoring and arterial blood gas analysis. An arterial blood gas sample obtained prior to incision revealed a PaO_2_ of 103 mmHg on an FiO_2_ of 0.5. Peak airway pressures, initially 38 cm H_2_O, progressively decreased to 17 cm H_2_O following gradual ascitic fluid drainage.

A total of 28 litres of ascitic fluid was drained slowly. A total of 100 mL of 4% albumin was administered intraoperatively to maintain colloid oncotic pressure. Total intraoperative fluids included 5000 mL crystalloids, 500 mL colloid, 100 mL albumin, and 700 mL packed red blood cells. Estimated blood loss was 1.5 litres, and urine output was 330 mL. The duration of surgery was approximately six hours.

Postoperatively, the patient required short-term continuous positive airway pressure (CPAP) support in the intensive care unit due to reduced respiratory effort and low tidal volumes. She was successfully extubated the following day. Postoperative analgesia was maintained using 0.125% bupivacaine via the epidural catheter every eight hours. Final histopathology confirmed mucinous cystadenocarcinoma of the ovary.

## Discussion

This case highlights the successful anaesthetic management of a patient with a giant ovarian tumour containing 28 litres of ascitic fluid without major perioperative complications. General anaesthesia combined with epidural analgesia was chosen as the patient had a normal coagulation profile, and epidural analgesia is known to reduce anaesthetic requirements, attenuate surgical stress responses, decrease systemic opioid use, and improve postoperative recovery [8].

Tracheal intubation was performed in the ramp position, aligning the external auditory meatus with the sternal notch, a technique recommended in obese patients to improve airway alignment, oxygenation, and ventilation while increasing safe apnoea time [9].

Massive ascites results in elevated intra-abdominal pressure and reduced functional residual capacity, predisposing patients to hypoxaemia and increased airway pressures [10]. Adequate preoxygenation and lung-protective ventilation strategies, including low tidal volumes, application of PEEP, and appropriate respiratory rates, were employed to maintain normocapnia and adequate oxygenation.

Gradual drainage of ascitic fluid was essential to prevent sudden haemodynamic collapse associated with rapid decompression. A balanced fluid replacement strategy was adopted to preserve preload, maintain colloid oncotic pressure, and ensure end-organ perfusion. Blood loss was replaced according to the calculated maximal allowable blood loss [11].

Central venous access was considered but deferred due to the absence of ultrasound guidance and concerns regarding worsened respiratory mechanics in Trendelenburg positioning. Haemodynamic stability was effectively maintained using large-bore peripheral access, invasive arterial monitoring, serial arterial blood gas analyses, and lactate measurements to assess tissue perfusion [12].

Active warming strategies, including forced-air warming blankets, warmed intravenous fluids, warmed irrigation solutions, and maintenance of ambient operating room temperature, were employed to prevent perioperative hypothermia [12].

## Conclusions

Anaesthetic management of patients with giant ovarian tumours and massive ascites is challenging due to significant respiratory compromise, haemodynamic instability, and major fluid shifts. Thorough preoperative evaluation, lung-protective ventilation strategies, invasive haemodynamic monitoring, and gradual ascitic fluid drainage are critical to minimise perioperative complications.

In this case, the combination of general anaesthesia and epidural analgesia provided optimal intraoperative stability and effective postoperative pain control. Careful postoperative monitoring and short-term ventilatory support contributed to a favourable outcome, underscoring the importance of meticulous planning and individualised perioperative management.
